# Social connection measures for older adults living in long-term care homes: a systematic review protocol

**DOI:** 10.1186/s13643-024-02468-6

**Published:** 2024-02-15

**Authors:** Madalena P. Liougas, Andrew Sommerlad, Hannah M. O’Rourke, Katherine S. McGilton, Jennifer Bethell

**Affiliations:** 1 Rehabilitation Sciences Institute, Temerty Faculty of Medicine, University of Toronto, 500 University Avenue, Suite 160, Toronto, ON Canada; 2 KITE Research Institute, Toronto Rehabilitation Institute-University Health Network, Toronto, ON Canada; 3 Division of Psychiatry, University College London, London, UK; 4 Camden and Islington NHS Foundation Trust, St Pancras Hospital, London, UK; 5 College of Health Sciences, Faculty of Nursing, University of Alberta, Alberta, Canada; 6 Lawrence S. Bloomberg Faculty of Nursing, University of Toronto, Toronto, ON Canada; 7 Institute of Health Policy, Management and Evaluation, University of Toronto, Toronto, ON Canada

**Keywords:** Long-term care, Measurement, Social connection

## Abstract

**Background:**

Various measures have assessed social connection in long-term care (LTC) home residents. However, they use inconsistent terminology, conceptualizations, and operationalizations of social connection. In this systematic review protocol, we propose a study that will characterize measures that assess aspects of LTC home residents’ social connection using a unified conceptual model. The objectives are to (1) describe and analyze the measures and (2) evaluate their measurement properties.

**Methods:**

A literature search was conducted in MEDLINE ALL (Ovid), Embase Classic and Embase (Ovid), Emcare Nursing (Ovid), APA PsycInfo (Ovid), Scopus, CINAHL Complete (EBSCOhost), AgeLine (EBSCOhost), and Sociological Abstracts (ProQuest). We will include primary research papers with no language limit, published from database inception. We will include studies of a measure of any aspect of social connection in LTC home residents that report at least one measurement property. Independently, two reviewers will screen titles and abstracts, review full-text articles against eligibility criteria, and extract data from included studies. In objective 1, we will analyze identified tools using an adapted framework method. In objective 2, we will evaluate each measure’s measurement properties using COnsensus-based Standards for the selection of health Measurement INstruments (COSMIN) methodology. We will engage experts and stakeholders to assist with interpreting results and translating knowledge.

**Discussion:**

Our findings will inform the social connection in long-term care home residents (SONNET) study’s development of a novel, person-centered measure for social connection in LTC home settings. We will present our findings in academic and non-academic forums, including conferences, peer-reviewed journals, and other publications.

**Systemic review registration:**

Prospero—“Systematic review of measures of social connection used in long-term care home research.” CRD42022303526.

**Supplementary Information:**

The online version contains supplementary material available at 10.1186/s13643-024-02468-6.

## Background

Social connection is an umbrella term that describes how people connect [1]; it is determined by the existence, roles, and sense of connection within our relationships [1, 2]. It includes observable and perceived aspects such as social isolation, loneliness, and social support, with associated considerations for measurement [3]. While evidence has demonstrated important associations between social connection and health [4–6], research in this area is limited by inconsistent terminology used to describe different aspects of social connection.

Valtorta et al. [7] proposed a novel, two-dimensional classification system for measures of social connection. In this framework, measures of social connection were distinguished by structure and function and by the degree of subjectivity [7]. However, it did not provide a conceptual model to describe how the aspects of social connection are related to each other, nor was it specific to long-term care (LTC) homes. Residents of LTC homes are mostly older adults [8, 9], and their relationships may affected by health status such as high prevalence of disability, chronic illness, and functional limitation [10] which also present considerations for measurement. Residents of LTC homes are isolated from their former social systems and are restricted in their ability to maintain relationships outside of the LTC home [11]. For example, dementia is common among LTC home residents [12], and residents with dementia can struggle to develop and maintain social connections, in part due to symptoms of dementia but also related to others’ lack of understanding and stigma towards dementia [11]. Similar to studies of older adults in other settings, poor social connection for LTC home residents is linked to worse mental and physical health [13, 14], including an increased risk of mortality [15], depression and anxiety [16], frailty [17], and cognitive decline [18]. Moreover, social connection is important to LTC home residents as it is a key concept for well-being, person-centered care delivery, and a sense of home [19–21].

Previous reviews have described evidence for interventions that address aspects of social connection in LTC home residents [10, 22, 23]. However, similar to broader social connection studies [7], there is little consensus on the best approaches to assessing social connection in LTC home residents. Measures used with LTC home residents may differ from other measures to account for characteristics of the residents and setting [24], such as approaches to ensure relevance, comprehensiveness, and comprehensibility as well as use of observer- or proxy-rated measures [25, 26].

**Fig. 1:**
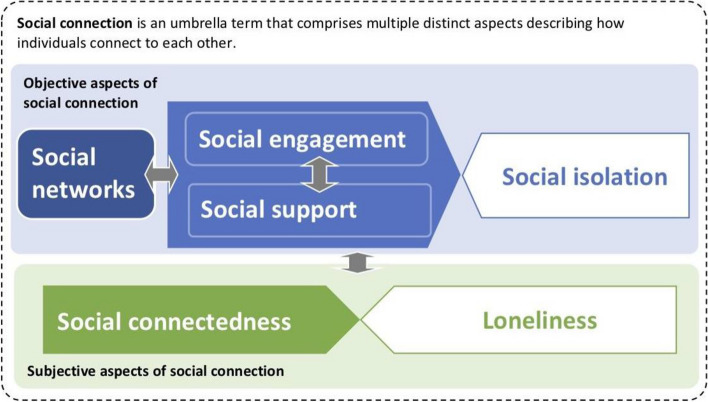
A preliminary conceptual model of social connection

Here, we offer a unifying conceptual model (see Fig. 1) to articulate how the aspects of social connection previously defined in the research literature are related within the LTC home context. This model for social connection uses concepts and definitions from a framework initially proposed by Berkman et al. [27] to explain the relationship between objective social connection and health—and then adapted for research in LTC homes by Leedahl et al. [28]. We build on these models by adding subjective aspects of social connection (i.e., loneliness [29, 30] and social connectedness [31]), objective social isolation [1], and representations of bidirectional associations between the different aspects of social connection. Objective and subjective aspects of social connection are related but distinct; for example, one can be objectively socially isolated but not feel lonely (and vice versa). Informed by our review’s findings, we will further elaborate and modify our conceptual model.

Aspects of social connection in LTC home residents have been assessed using various measures, including dedicated scales and subscales in measures capturing general health perceptions such as quality of life [10, 32]. Although different approaches to measurement in LTC home settings have been described [32], existing reviews have not addressed how measures operationalize the distinct aspects of social connection or review their measurement properties. Furthermore, it is unclear if and how these measures and their operationalization of social connection overlap, how dementia-specific measures differ from those for broader use, and the quality of each measure. This review aims to characterize measures tested in LTC home settings that assess any aspect of residents’ social connection. In objective 1, we will describe these measures according to the aspects of social connection assessed and analyze how dementia-specific measures may differ from those developed for broader use among all LTC home residents. In objective 2, we will evaluate the measurement properties of the measures.

This protocol outlines the processes in aim one of the social connection in long-term care home residents (SONNET) study. This aim consists of two distinct objectives. Both objectives will use the same eligibility criteria, patient and public involvement and engagement, and search strategy. However, they are distinguished from one another in the data extraction and data analysis sections.

We conducted a preliminary search of MEDLINE, the Cochrane Database of Systematic Reviews, Open Science Framework, and JBI Evidence Synthesis. Madrigal et al. [32] addressed measuring social functioning in nursing home residents. However, no existing systematic reviews address measuring social connection in LTC home residents.

### Review questions

Objective 1: How is social connection operationalized for measurement in LTC home residents? How do dementia-specific measures differ from those developed for broader use among all LTC home residents?

Objective 2: What are the measurement properties and overall quality of the measures that have assessed social connection in LTC home residents?

## Methods

Objectives 1 and 2 will follow the Joanna Briggs Institute (JBI) review methods [33]. The protocol was registered on PROSPERO in January 2021. Any changes or updates to the protocol (e.g., eligibility criteria, search strategy, data extraction, or data analysis) will be documented in the final manuscripts.

### Eligibility criteria

#### Population: older adults

Studies of older adults, with or without cognitive impairment or dementia. The focus is on older adults as they comprise the majority of LTC residents [8, 9]; studies will not be restricted to only those of older adults. However, studies will only be included if the mean age of participants is 65 years or older (or at least 2/3 of participants are 65 years or older).

#### Concept: social connection

Studies of measures assessing any objective or subjective aspects of social connection (Fig. 1), defined according to working definitions provided in Additional file 1: Appendix I [1, 27–31, 34]. Measures with subscales and items assessing aspects of social connection will be included if these subscales/items are reported separately. However, studies of measures that provide only the summary scores of other concepts (e.g., overall quality of life) will be excluded.

#### Context: LTC homes

Populations must reside in a LTC home setting defined according to the international definition of a nursing home [35]. Studies must report that at least 2/3 of participants were LTC home residents or present results for LTC home residents separately. Studies conducted exclusively in other congregate settings (e.g., assisted living, hospice, independent living, retirement homes) will be excluded.

### Types of sources

This review will consider primary research publications with no language restrictions. Relevant reviews will also be scanned for eligible publications. Consistent with COSMIN recommendations, secondary texts, literature reviews, conference abstracts, editorials, and dissertations will be excluded as they do not have sufficient detail regarding study design. We worked with an experienced information specialist to develop comprehensive, inclusive search strategies that were employed in multiple bibliographic databases (see Additional file 2: Appendix II). Grey literature will not be included.

### Search strategy (objectives 1 and 2)

The search strategy was adapted from a previous study [36] and updated by an experienced information specialist (see Additional file 2: Appendix II). Electronic databases that will be searched include MEDLINE ALL (Ovid), Embase Classic and Embase (Ovid), Emcare Nursing (Ovid), APA PsycInfo (Ovid), Scopus, CINAHL Complete (EBSCOhost), AgeLine (EBSCOhost), and Sociological Abstracts (ProQuest). Databases will be searched for published research studies that quantify any aspect of social connection that has been identified for use in research in LTC homes. The working definitions of the key aspects of social connection, relevant to the conceptual model (Fig. 1), are provided in Additional file 1: Appendix I. A broad range of search terms will be used to acknowledge relevant studies that might use alternative terminology. To meet the context requirements of this study, terms including long-term care, nursing home, care home, residential home, and home for the elderly will be used to reflect the variance in terminology sometimes used to describe LTC homes [35]. To search for studies that report measurement properties, the COSMIN filters, which have a sensitivity of 97.4% and a precision (akin to positive predictive value) of 4.4%, will be applied [37]. We will also scan reference lists of included studies and contact experts to seek additional eligible studies.

### Study/source of evidence selection (objectives 1 and 2)

Citations will be exported from each database, uploaded to Endnote X9.1 (Clarivate Analytics, PA, USA), and imported into Covidence for duplicate removal. A pilot test screening for 15 papers (titles and abstracts) will be conducted to familiarize reviewers with eligibility criteria. Following the pilot test, titles and abstracts will be screened by two reviewers. For full-text review, publications will be imported into Covidence and assessed by two reviewers. Non-English papers will be assessed by additional reviewers with relevant language and research expertise. Reasons for exclusion will be stated as (1) types of sources (e.g., no measurement properties reported), (2) setting: not LTC, (3) concept: not social connection, and (4) population (e.g., not older adults, not LTC residents). Reasons for exclusion will be recorded in Covidence. Any disagreements that arise throughout the screening of abstracts/titles or full text will be resolved through a discussion with a third reviewer. The results of the search and study eligibility will be reported in the final review and presented in a Preferred Reporting Items for Systematic Reviews and Meta-analyses (PRISMA) flow diagram [33]. We used the PRISMA-P checklist for this protocol [38].

### Data extraction (objectives 1 and 2)

Data will be extracted independently by two researchers using standardized data extraction instructions.

For each included study, the data extraction will include population (country, race/ethnicity, inclusion criteria, exclusion criteria, sample size—number of residents and homes, gender/sex, age), social connection concept, name of measure, response options, mode of administration, and observation period. A draft of the extraction form is provided in Additional file 3: Appendix III. The extraction form will be modified as necessary during data extraction and analysis. Modifications will be detailed in the final reviews. Any disagreements between reviewers will be resolved through discussion or by a third reviewer.

#### Objective 1

Additional data that will be extracted are the individual items (questions and statements) that are used to assess social connection.

#### Objective 2

Data will be extracted using the predefined data collection template provided by COSMIN (available at www.cosmin.nl). This includes data on each measure’s measurement properties (i.e., content validity, structural validity, internal consistency, cross-cultural validity/measurement invariance, reliability, measurement error, criterion validity, construct validity, and responsiveness).

### Data analysis

Selected studies and measures will be summarized in tables reporting frequency and percentage statistics to describe the studies (author, year of publication, population) and measure (social connection aspects, name of measure, response options, mode of administration, and observation period).

#### Objective 1

Objective 1 will be analyzed using an adapted framework method [39].

**Stage 1.1: transcription**

A copy of each measure will be obtained from the original study, online search, or contact with study authors. Studies that report on the same measure will be grouped; measures will be identified as dementia-specific or generic (non-dementia-specific). The name of each measure and the terms it uses to describe social connection will be identified*.*

**Stage 1.2: familiarization**

The items within each measure will be reviewed. Thoughts and impressions concerning how items describe social connection aspects will be documented in a journal. Familiar aspects of social connection (Fig. 1) will have their items reviewed alongside the working definitions (Additional file 1: Appendix I). Unfamiliar aspects of social connection will have their items reviewed so that a working definition can be created. Thoughts and impressions on how these unfamiliar aspects fit into the preliminary conceptual model (Fig. 1) will be recorded in a journal.

**Stage 1.3: coding**

A hybrid deductive-inductive coding approach will be used [40] where codes are based on the working definitions of social connection aspects [39]. Using a sample of five to ten randomly selected measures, two researchers will code the items in these measures separately.

**Stage 1.4: developing a working analytical framework**

Following coding of the sample measures, researchers will meet and compare the coding labels. Researchers will agree on a set of codes to apply to subsequent measures. Codes may be grouped together if both researchers agree their working definitions overlap. Any disagreements will be resolved through discussion with the wider research team. The preliminary conceptual model for social connection (Fig. 1) will be revised to include any new aspects of social connection. This revised conceptual model will form an analytical framework for social connection in measurement.

**Stage 1.5: applying the analytical framework**

Each measure’s items will be sorted into the analytical framework for social connection in measurement. An electronic software such as Computer Assisted Qualitative Data Analysis Software (CAQDAS) may be used to complete this process.

**Stage 1.6: charting the data into the framework matrix**

The data from the analytical framework will be used to generate a matrix (table) whereby the rows represent “cases” (measures) and the columns represent the codes (aspects of social connection) developed in stage 1.4. The items in each measure will be charted into the appropriate column (i.e., according to the aspect of social connection).

**Stage 1.7: interpreting the data**

Data will be interpreted in three steps. First, each measure will be summarized according to the codes to which it was mapped; dementia and non-dementia-specific measures will be compared by tabulating the presence of codes. Next, inductive coding will be applied to identify themes within codes and compare them across dementia and non-dementia-specific measures.

#### Objective 2

Objective 2 will be conducted and reported following the COnsensus-based Standards for the selection of health Measurement Instruments (COSMIN) guidelines [41]. COSMIN outlines rigorous, transparent methodology and standards used to assess measurement properties, feasibility, and interpretability of a measure, and the methodological quality of the studies which report measurement properties.

**Stage 2.1: assessment of measurement properties**

Two researchers will extract data on each study’s measurement properties, with measurement properties being defined using the COSMIN taxonomy [42], including content validity, structural validity, internal consistency, cross-cultural validity, reliability, measurement error, criterion validity, construct validity, and responsiveness.

Measurement properties will be rated as sufficient ( +), insufficient ( −), or indeterminate (?) [26]. COSMIN’s [43] modified criteria for good measurement properties (adapted from Terwee et al. [44] (Additional file 4: Appendix IV) will be used to provide evidence of the quality of measurement properties.

**Stage 2.2: evaluate measurement properties**

Two researchers will independently and systematically evaluate each measure of social connection by providing summarized results of each measure’s measurement properties. Completed versions of the COSMIN risk of bias checklists on patient-reported outcome measure development, content validity, structural validity, internal consistency, cross-cultural validity/measurement invariance, reliability, measurement error, criterion validity, hypotheses testing for construct validity, and responsiveness will be used to compute the overall ratings for summarized results. Overall ratings for these results will be labeled as sufficient ( +), insufficient ( −), or indeterminate (?) (Additional file 4: Appendix IV).

**Stage 2.3: grading the quality of the evidence**

The research team will summarize the evidence for each measure (and each measurement property) and provide an overall rating using a modified Grading of Recommendations Assessment, Development, and Evaluation (GRADE) approach [45]. This approach grades the quality of the evidence as high, moderate, low, or very low.

### Patient and public involvement (objectives 1 and 2)

During this review, people living with dementia and friends, family or current/former caregivers of people with dementia and/or in LTC homes will be involved through the Canadian Consortium on Neurodegeneration in Aging’s Engagement of People with Lived Experience of Dementia (EPLED) program (www.epled.ca) [46] and the Alzheimer’s Society (UK) Research Network. During the preparatory phase [47], EPLED members were involved in identifying the research topic. During the execution phase, people with lived experience will contribute to interpreting results and knowledge translation. In particular, preliminary results will be presented to panel members in workshops where they will be asked to provide feedback (oral or written) and assist in identifying relevant themes and patterns surrounding important aspects of social connection. As part of the SONNET study, people with lived experience will also assist in knowledge translation by disseminating results to academic and non-academic audiences.

## Discussion

This systematic review will provide an inventory of existing measures of social connection in LTC home populations. In objective 1, we will describe the aspects of social connection assessed by each measure. Inconsistent conceptual terminology used within measures will be clarified by mapping each measure’s items to the aspects in our unified conceptual model, namely, social network [27, 28], social engagement [27, 28], social support [34], social isolation [1], social connectedness [31], and loneliness [29, 30]. In objective 2, we will present evidence and recommendations for the future use of measures based on their measurement properties: content validity, structural validity, internal consistency, cross-cultural validity, reliability, measurement error, criterion validity, construct validity, and responsiveness [42, 48]. Recommendations for measures will be classified into one of the three following categories: “A,” “B,” or “C” [41]. Measures categorized as “A” will be recommended for use as they will have evidence of sufficient content validity and at least low-quality evidence for sufficient internal consistency. Measures categorized as “B” will potentially be recommended for use, but they will require further research to assess the quality of the measure. Measures categorized as “C” will not be recommended for use as they will have high-quality evidence of an insufficient measurement property. The SONNET team will use the results from this review to identify gaps in the content and quality of existing measures that assess LTC home residents’ social connections, demonstrating the need to create a novel measure. Our review will also inform future measure selection by highlighting the aspects and measurement properties of each social connection measure. This is important for furthering the evidence on how social connection affects health and in identifying effective interventions targeting social connection for LTC home residents.

Our findings will be presented in academic and non-academic forums, including conferences, peer-reviewed journals, and other publications. People with lived experience of dementia and LTC homes will be engaged in contextualizing findings and knowledge translation. To our knowledge, this is the first systematic review of measures of social connection assessed in LTC home residents. Despite these strengths, our review will be limited by our eligibility criteria that focus exclusively on measures that have undergone formal psychometric analysis in LTC home settings. By only including peer-reviewed literature, we anticipate that identified studies will be of greater methodological quality and measures are more likely to have undergone rigorous psychometric analysis [49]. However, we acknowledge the potential for incomplete reporting of measures, study methods, and measurement property results in published studies. We will discuss this limitation in context with study findings.

### Supplementary Information

**Additional file 1: Appendix I.**

**Additional file 2: Appendix II.**

**Additional file 3: Appendix III.**

**Additional file 4: Appendix IV.**

## Data Availability

Not applicable as no data or statistical code is available. The list of studies and measures will be published as part of the reporting.

## Abbreviations

LTC: Long-term care
SONNET: Social connection in long-term care home residents
COSMIN: COnsensus-based Standards for the selection of health Measurement INstruments
