# Supplementary material for: Social connection measures for older adults living in long-term care homes: a systematic review protocol
Source: Syst Rev. 2024 Feb 15;13:67. doi: 10.1186/s13643-024-02468-6 (PMC10867987; doi:10.1186/s13643-024-02468-6)
Supplement: Supplementary file 1 — Additional file 1: Appendix I. [file 13643_2024_2468_MOESM1_ESM.pdf]

Appendix I

Table 1. "Working definitions" of social connection and the aspects that are used to describe social connection

	**Working Definition**
Social Connection	An umbrella term that comprises multiple distinct aspects describing how individuals connect to each other (1).<br>It depends on the existence, roles and qualities of relationships as well as the sense of connection in these relationships.
**Objective Aspects**	
Social Networks	Web of social relationships that surround an individual and the characteristics of those ties (3,4).
Social Engagement	Taking part in activities within the communities in which people live.<br>May include:<br>• productive activities<br>• social activities<br>• leisure activities (3,4).
Social Support	exchange of resources between at least two individuals intended to enhance the well-being of the recipient. May include:<br>• emotional (expressions of empathy, love, trust, caring)<br>• instrumental (tangible help)<br>• informational (advice, suggestions, information)<br>• appraisal (information for self-evaluation) (5).
Social Isolation	The objective lack of (or limited) social contact with others (1).
**Subjective Aspects**	
Social Connectedness	The extent to which one has meaningful, close, and constructive relationships with others; opposite of loneliness (6).
Loneliness	Negative experience resulting from the discrepancy between an individual's desired and actual experience of meaningful connections. May include:<br>• emotional loneliness (lack of a close intimate attachment to another person, feeling isolated or alone)<br>• social loneliness (lack of a social network, feeling left out) (7,8).
**Related Aspects**	

Social Capital	Value of social networks for influencing productivity of individuals and groups creating economic gain (3).
Social Interaction	The quality of the verbal and non-verbal behaviors that are exercised between an individual and others in their surroundings.
Social Participation	Interacting and sharing resources with other people in the community (9).

References

1. Committee on the Health and Medical Dimensions of Social Isolation and Loneliness in Older Adults, Board on Health Sciences Policy, Board on Behavioral, Cognitive, and Sensory Sciences, Health and Medicine Division, Division of Behavioral and Social Sciences and Education, National Academies of Sciences, Engineering, and Medicine. National Academies of Sciences E and M. Social Isolation and Loneliness in Older Adults: Opportunities for the Health Care System [Internet]. Washington, D.C.: National Academies Press; 2020 [cited 2022 Mar 14]. Available from: https://www.nap.edu/catalog/25663
2. Holt-Lunstad J. Why Social Relationships Are Important for Physical Health: A Systems Approach to Understanding and Modifying Risk and Protection. Annu Rev Psychol. 2018 Jan 4;69(1):437–58.
3. Leedahl SN, Sellon A, Chapin RK. Assessment of multiple constructs of social integration for older adults living in nursing homes. J Gerontol Soc Work. 2018 Jul 4;61(5):526–48.
4. Berkman LF, Glass T, Brissette I, Seeman TE. From social integration to health: Durkheim in the new millennium. Soc Sci Med. 2000 Sep;51(6):843–57.
5. House JS. Work stress and social support. Reading, Mass: Addison-Wesley Pub. Co; 1981. 156 p. (Addison-Wesley series on occupational stress).
6. O'Rourke HM, Sidani S. Definition, Determinants, and Outcomes of Social Connectedness for Older Adults: A Scoping Review. J Gerontol Nurs. 2017 Jul;43(7):43–52.
7. Prohaska T, Burholt V, Burns A, Golden J, Hawkley L, Lawlor B, et al. Consensus statement: loneliness in older adults, the 21st century social determinant of health? BMJ Open. 2020 Aug;10(8):e034967.
8. Weiss RS. Loneliness: the experience of emotional and social isolation. Cambridge, Mass: MIT Press; 1974. 236 p.
9. Dehi M, Mohammadi F. Social Participation of Older Adults: A Concept Analysis. Int J Community Based Nurs Midwifery [Internet]. 2020 Jan [cited 2022 Feb 22];8(1). Available from: https://doi.org/10.30476/ijcbnm.2019.82222.1055
