# Supplementary material for: Social connection measures for older adults living in long-term care homes: a systematic review protocol
Source: Syst Rev. 2024 Feb 15;13:67. doi: 10.1186/s13643-024-02468-6 (PMC10867987; doi:10.1186/s13643-024-02468-6)
Supplement: Supplementary file 2 — Additional file 2: Appendix II. [file 13643_2024_2468_MOESM2_ESM.pdf]

Appendix II: Search Strategy

Search strategy designed with an Information Specialist.

“Electronic databases that were searched include: MEDLINE ALL (Ovid), Embase Classic and Embase (Ovid), Emcare Nursing (Ovid), APA PsycInfo (Ovid), Scopus, CINAHL Complete (EBSCOhost), AgeLine (EBSCOhost), and Sociological Abstracts (ProQuest). The search strategy was originally developed in Medline, and subsequently translated to all other databases. The search strategy consisted of two concepts and one search filter combined using the Boolean operator “AND”. The search strategies for the concepts of (1) social connection and (2) LTC, nursing homes, or homes for the aged were sourced from a previous review and used without modifications (2). The highly sensitive COSMIN filter was used without modifications (33). Non-validated translations were used when available. The COSMIN filter was used to identify studies on measurement properties with a sensitivity of 97.4% and a precision of 4.4% (24). Limits were applied to focus on human adult studies and to exclude conference proceedings or limit to

Total Records (PRISMA Flowchart -November 2021 numbers)
· MEDLINE ALL (Ovid): 1995 Results
· Embase Classic and Embase (Ovid): 1553 Results
· Emcare Nursing (Ovid): 1764 Results
· APA PsycInfo (Ovid): 619 Results
· Scopus: 476 Results
· CINAHL Complete (EBSCOhost): 502 Results
· AgeLine(EBSCOhost): 280 Results
· ProQuest Sociological Abstracts (ProQuest): 33 Results
Total: 7222 Results

Database: Ovid MEDLINE(R) ALL <1946 to November 16, 2021>
Interface: Ovid
Latest search date: November 17 2021
1 Interpersonal Relations/
2 exp social support/
3 Social Participation/
4 exp Social Isolation/
5 Social inclusion/
6 Social Interaction/
7 Social Network Analysis/
8 Loneliness/

9 Social Behavior/

10 ((social or socially) adj3 (engaged or engagement)).tw,kf.

11 ((social or socially) adj3 (disengaged or disengagement)).tw,kf.

12 ((social or socially) adj3 (connect* or disconnect*)).tw,kf.

13 Sociali?ation.tw,kf.

14 Sociali??.tw,kf.

15 sociali?ing.tw,kf.

16 Interpersonal relation?.tw,kf.

17 (social adj3 behavio?r*).tw,kf.

18 (social adj3 capital?).tw,kf.

19 ((social or socially) adj3 support??).tw,kf.

20 (social adj3 relationship?).tw,kf.

21 ((social or socially) adj3 participat*).tw,kf.

22 (social adj3 network?).tw,kf.

23 (social adj3 interaction*).tw,kf.

24 (personal adj3 network?).tw,kf.

25 friendship?.tw,kf.

26 ((social or socially) adj3 integrat*).tw,kf.

27 (social adj3 relation?).tw,kf.

28 (social adj3 contact).tw,kf.

29 ((social or socially) adj3 embedded*).tw,kf.

30 lonel*.tw,kf.

31 ((social or socially) adj3 isolat*).tw,kf.

32 (emotional* adj3 isolat*).tw,kf.

33 ((perceive* or percept*) adj3 isolat*).tw,kf.

34 ((social* or socially) adj3 alienat*).tw,kf.

35 aloneness.tw,kf.

36 solitud*.tw,kf.

37 Unwanted alone.tw,kf.

38 or/1-37

39 Nursing Homes/

40 Residential Facilities/

41 Homes for the Aged/

42 Long-Term Care/

43 "long-term care".tw,kf.

44 "longterm care".tw,kf.

45 LTC.tw,kf.

46 ((home? or facility or facilities) adj2 (retirement or assisted living or residential care or extended care)).tw,kf.

47 home? for the aged.tw,kf.

48 home? for the elderly.tw,kf.
49 old age home?.tw,kf.
50 ("old age facility" or "old age facilities").tw,kf.
51 (nursing adj3 (facility or facilities or residence? or center? or centre?)).tw,kf.
52 nursing home?.tw,kf.
53 (residence? adj2 ('assisted living' or convalescen* or retire???? or 'long stay' or longstay or 'long term')).tw,kf.
54 ((facility or facilities) adj2 ('assisted living' or convalescen* or retire???? or resident* or 'long stay' or longstay or 'long term')).tw,kf.
55 (home? adj2 ('assisted living' or convalescen* or retire???? or 'long stay' or longstay or 'long term')).tw,kf.
56 "care home?".tw,kf.
57 (housing adj3 (retirement or old age or senior* or elderly)).tw,kf.
58 or/39-57
59 38 and 58
60 (instrumentation or methods).fs.
61 (Validation Studies or Comparative Study).pt.
62 exp Psychometrics/
63 psychometr*.ti,ab.
64 (clinimetr* or clinometr*).tw.
65 exp "Outcome Assessment (Health Care)"/
66 outcome assessment.ti,ab.
67 outcome measure*.tw.
68 exp Observer Variation/
69 observer variation.ti,ab.
70 exp Health Status Indicators/
71 exp "Reproducibility of Results"/
72 reproducib*.ti,ab.
73 exp Discriminant Analysis/
74 (reliab* or unreliab* or valid* or coefficient or homogeneity or homogeneous or "internal consistency").ti,ab.
75 (cronbach* and (alpha or alphas)).ti,ab.
76 (item and (correlation* or selection* or reduction*)).ti,ab.
77 (agreement or precision or imprecision or "precise values" or test-retest).ti,ab.
78 (test and retest).ti,ab.
79 (reliab* and (test or retest)).ti,ab.
80 (stability or interrater or inter-rater or intrarater or intra-rater or intertester or inter-tester or intratester or intra-tester or interobserver or inter-observer or intraobserver or intraobserver or intertechnician or inter-technician or intratechnician or intra-technician or interexaminer or inter-examiner or intraexaminer or intra-examiner or interassay or interassay or intraassay or intra-

assay or interindividual or inter-individual or intraindividual or intra-individual or interparticipant or inter-participant or intraparticipant or intra-participant or kappa or kappa's or kappas or repeatab*).ti,ab.

81 ((replicab* or repeated) and (measure or measures or findings or result or results or test or tests)).ti,ab.

82 (generaliza* or generalisa* or concordance).ti,ab.

83 (intraclass and correlation*).ti,ab.

84 (discriminative or "known group" or factor analysis or factor analyses or dimension* or subscale*).ti,ab.

85 (multitrait and scaling and (analysis or analyses)).ti,ab.

86 (item discriminant or interscale correlation* or error or errors or "individual variability").ti,ab.

87 (variability and (analysis or values)).ti,ab.

88 (uncertainty and (measurement or measuring)).ti,ab.

89 ("standard error of measurement" or sensitiv* or responsive*).ti,ab.

90 ((minimal or minimally or clinical or clinically) and (important or significant or detectable) and (change or difference)).ti,ab.

91 (small* and (real or detectable) and (change or difference)).ti,ab.

92 (meaningful change or "ceiling effect" or "floor effect" or "Item response model" or IRT or Rasch or "Differential item functioning" or DIF or "computer adaptive testing" or "item bank" or "cross-cultural equivalence").ti,ab.

93 or/60-92

94 59 and 93

95 94 not ((exp infant/ or exp child/) not exp adult/)

96 95 not (exp animals/ not exp humans/)

Database: Embase Classic+Embase <1947 to 2021 November 17>

Interface: Ovid

Latest search date: November 18 2021

1 human relation/

2 social support/

3 social participation/

4 social interaction/

5 social capital/

6 social isolation/

7 loneliness/

8 social behavior/

9 ((social or socially) adj3 (engaged or engagement)).tw,kw.

10 ((social or socially) adj3 (disengaged or disengagement)).tw,kw.

11 ((social or socially) adj3 (connect* or disconnect*)).tw,kw.

12 Sociali?ation.tw,kw.
13 Sociali??.tw,kw.
14 sociali?ing.tw,kw.
15 Interpersonal relation?.tw,kw.
16 (social adj3 behavio?r*).tw,kw.
17 (social adj3 capital?).tw,kw.
18 ((social or socially) adj3 support??).tw,kw.
19 (social adj3 relationship?).tw,kw.
20 ((social or socially) adj3 participat*).tw,kw.
21 (social adj3 network?).tw,kw.
22 (social adj3 interaction*).tw,kw.
23 (personal adj3 network?).tw,kw.
24 friendship?.tw,kw.
25 ((social or socially) adj3 integrat*).tw,kw.
26 (social adj3 relation?).tw,kw.
27 (social adj3 contact).tw,kw.
28 ((social or socially) adj3 embedded*).tw,kw.
29 lonel*.tw,kw.
30 ((social or socially) adj3 isolat*).tw,kw.
31 (emotional* adj3 isolat*).tw,kw.
32 ((perceive* or percept*) adj3 isolat*).tw,kw.
33 ((social* or socially) adj3 alienat*).tw,kw.
34 aloneness.tw,kw.
35 solitud*.tw,kw.
36 Unwanted alone.tw,kw.
37 or/1-36
38 nursing home/
39 residential home/
40 home for the aged/
41 long term care/
42 "long-term care".tw,kw.
43 "longterm care".tw,kw.
44 LTC.tw,kw.
45 ((home? or facility or facilities) adj2 (retirement or assisted living or residential care or extended care)).tw,kw.
46 home? for the aged.tw,kw.
47 home? for the elderly.tw,kw.
48 old age home?.tw,kw.
49 ("old age facility" or "old age facilities").tw,kw.
50 (nursing adj3 (facility or facilities or residence? or center? or centre?)).tw,kw.

51 nursing home?.tw,kw.
52 (residence? adj2 ('assisted living' or convalescen* or retire???? or 'long stay' or longstay or 'long term')).tw,kw.
53 ((facility or facilities) adj2 ('assisted living' or convalescen* or retire???? or resident* or 'long stay' or longstay or 'long term')).tw,kw.
54 (home? adj2 ('assisted living' or convalescen* or retire???? or 'long stay' or longstay or 'long term')).tw,kw.
55 "care home?".tw,kw.
56 (housing adj3 (retirement or old age or senior* or elderly)).tw,kw.
57 or/38-56
58 37 and 57
59 exp "intermethod comparison"/
60 exp "data collection method"/
61 exp "validation study"/
62 exp "feasibility study"/
63 exp "pilot study"/
64 exp "psychometry"/
65 exp "reproducibility"/
66 reproducib*.ab,ti.
67 audit.ab,ti.
68 psychometr*.ab,ti.
69 clinimetr*.ab,ti.
70 clinometr*.ab,ti.
71 exp "observer variation"/
72 "observer variation".ab,ti.
73 exp "discriminant analysis"/
74 exp "validity"/
75 reliab*.ab,ti.
76 valid*.ab,ti.
77 coefficient.ab,ti.
78 "internal consistency".ab,ti.
79 (cronbach* and ("alpha" or "alphas")).ab,ti.
80 (item and (correlation* or selection* or reduction*)).ab,ti.
81 (agreement or precision or imprecision or "precise values" or test-retest).ab,ti.
82 (test and retest).ab,ti.
83 (reliab* and (test or retest)).ab,ti.
84 (stability or interrater or inter-rater or intrarater or intra-rater or intertester or inter-tester or intratester or intra-tester or interobserver or inter-observer or intraobserver or intraobserver or intertechnician or inter-technician or intratechnician or intra-technician or interexaminer or inter-examiner or intraexaminer or intra-examiner or interassay or interassay or intraassay or intra-

assay or interindividual or inter-individual or intraindividual or intra-individual or interparticipant or inter-participant or intraparticipant or intra-participant or kappa or kappa's or kappas or repeatab*).ab,ti.
85 ((replicab* or repeated) and (measure or measures or findings or result or results or test or tests)).ab,ti.
86 (generaliza* or generalisa* or concordance).ab,ti.
87 (intraclass and correlation*).ab,ti.
88 (discriminative or "known group" or factor analysis or factor analyses or dimension* or subscale*).ab,ti.
89 (multitrait and scaling and (analysis or analyses)).ab,ti.
90 (item discriminant or interscale correlation* or error or errors or "individual variability").ab,ti.
91 (variability and (analysis or values)).ab,ti.
92 (uncertainty and (measurement or measuring)).ab,ti.
93 ("standard error of measurement" or sensitiv* or responsive*).ab,ti.
94 ((minimal or minimally or clinical or clinically) and (important or significant or detectable) and (change or difference)).ab,ti.
95 (small* and (real or detectable) and (change or difference)).ab,ti.
96 (meaningful change or "ceiling effect" or "floor effect" or "Item response model" or IRT or Rasch or "Differential item functioning" or DIF or "computer adaptive testing" or "item bank" or "cross-cultural equivalence").ab,ti.
97 or/59-96
98 58 and 97
99 98 not ((exp embryo/ or exp fetus/ or exp juvenile/) not exp adult/)
100 99 not ((exp animals/ or exp animal experimentation/ or nonhuman/) not exp human/)
101 limit 100 to (conference abstract or conference paper or "conference review")
102 100 not 101
103 102 not medline.cr.

Database: Ovid Emcare Nursing <1995 to Present>

Interface: Ovid

Latest search date: November 18 2021

1 human relation/
2 social support/
3 social participation/
4 social interaction/
5 social capital/
6 social isolation/
7 loneliness/
8 social behavior/

9 ((social or socially) adj3 (engaged or engagement)).tw,kw.
10 ((social or socially) adj3 (disengaged or disengagement)).tw,kw.
11 ((social or socially) adj3 (connect* or disconnect*)).tw,kw.
12 Sociali?ation.tw,kw.
13 Sociali??.tw,kw.
14 sociali?ing.tw,kw.
15 Interpersonal relation?.tw,kw.
16 (social adj3 behavio?r*).tw,kw.
17 (social adj3 capital?).tw,kw.
18 ((social or socially) adj3 support??).tw,kw.
19 (social adj3 relationship?).tw,kw.
20 ((social or socially) adj3 participat*).tw,kw.
21 (social adj3 network?).tw,kw.
22 (social adj3 interaction*).tw,kw.
23 (personal adj3 network?).tw,kw.
24 friendship?.tw,kw.
25 ((social or socially) adj3 integrat*).tw,kw.
26 (social adj3 relation?).tw,kw.
27 (social adj3 contact).tw,kw.
28 ((social or socially) adj3 embedded*).tw,kw.
29 lonel*.tw,kw.
30 ((social or socially) adj3 isolat*).tw,kw.
31 (emotional* adj3 isolat*).tw,kw.
32 ((perceive* or percept*) adj3 isolat*).tw,kw.
33 ((social* or socially) adj3 alienat*).tw,kw.
34 aloneness.tw,kw.
35 solitud*.tw,kw.
36 Unwanted alone.tw,kw.
37 or/1-36
38 nursing home/
39 residential home/
40 home for the aged/
41 long term care/
42 "long-term care".tw,kw.
43 "longterm care".tw,kw.
44 LTC.tw,kw.
45 ((home? or facility or facilities) adj2 (retirement or assisted living or residential care or extended care)).tw,kw.
46 home? for the aged.tw,kw.
47 home? for the elderly.tw,kw.

48 old age home?.tw,kw.
49 ("old age facility" or "old age facilities").tw,kw.
50 (nursing adj3 (facility or facilities or residence? or center? or centre?)).tw,kw.
51 nursing home?.tw,kw.
52 (residence? adj2 ('assisted living' or convalescen* or retire???? or 'long stay' or longstay or 'long term')).tw,kw.
53 ((facility or facilities) adj2 ('assisted living' or convalescen* or retire???? or resident* or 'long stay' or longstay or 'long term')).tw,kw.
54 (home? adj2 ('assisted living' or convalescen* or retire???? or 'long stay' or longstay or 'long term')).tw,kw.
55 "care home?".tw,kw.
56 (housing adj3 (retirement or old age or senior* or elderly)).tw,kw.
57 or/38-56
58 37 and 57
59 exp "intermethod comparison"/
60 exp "data collection method"/
61 exp "validation study"/
62 exp "feasibility study"/
63 exp "pilot study"/
64 exp "psychometry"/
65 exp "reproducibility"/
66 reproducib*.ab,ti.
67 audit.ab,ti.
68 psychometr*.ab,ti.
69 clinimetr*.ab,ti.
70 clinometr*.ab,ti.
71 exp "observer variation"/
72 "observer variation".ab,ti.
73 exp "discriminant analysis"/
74 exp "validity"/
75 reliab*.ab,ti.
76 valid*.ab,ti.
77 coefficient.ab,ti.
78 "internal consistency".ab,ti.
79 (cronbach* and ("alpha" or "alphas")).ab,ti.
80 (item and (correlation* or selection* or reduction*)).ab,ti.
81 (agreement or precision or imprecision or "precise values" or test-retest).ab,ti.
82 (test and retest).ab,ti.
83 (reliab* and (test or retest)).ab,ti.

84 (stability or interrater or inter-rater or intrarater or intra-rater or intertester or inter-tester or intratester or intra-tester or interobserver or inter-observer or intraobserver or intraobserver or intertechnician or inter-technician or intratechnician or intra-technician or interexaminer or inter-examiner or intraexaminer or intra-examiner or interassay or interassay or intraassay or intra-assay or interindividual or inter-individual or intraindividual or intra-individual or interparticipant or inter-participant or intraparticipant or intra-participant or kappa or kappa's or kappas or repeatab*).ab,ti.

85 ((replicab* or repeated) and (measure or measures or findings or result or results or test or tests)).ab,ti.

86 (generaliza* or generalisa* or concordance).ab,ti.

87 (intraclass and correlation*).ab,ti.

88 (discriminative or "known group" or factor analysis or factor analyses or dimension* or subscale*).ab,ti.

89 (multitrait and scaling and (analysis or analyses)).ab,ti.

90 (item discriminant or interscale correlation* or error or errors or "individual variability").ab,ti.

91 (variability and (analysis or values)).ab,ti.

92 (uncertainty and (measurement or measuring)).ab,ti.

93 ("standard error of measurement" or sensitiv* or responsive*).ab,ti.

94 ((minimal or minimally or clinical or clinically) and (important or significant or detectable) and (change or difference)).ab,ti.

95 (small* and (real or detectable) and (change or difference)).ab,ti.

96 (meaningful change or "ceiling effect" or "floor effect" or "Item response model" or IRT or Rasch or "Differential item functioning" or DIF or "computer adaptive testing" or "item bank" or "cross-cultural equivalence").ab,ti.

97 or/59-96

98 58 and 97

99 98 not ((exp embryo/ or exp fetus/ or exp juvenile/) not exp adult/)

100 99 not ((exp animals/ or exp animal experimentation/ or nonhuman/) not exp human/)

101 limit 100 to (conference abstract or conference paper or "conference review")

102 100 not 101

103 102 not medline.cr.

Database: APA PsycInfo <1806 to November Week 3 2021>

Interface: Ovid

Latest search date: November 18 2021

1 interpersonal relationships/

2 social support/

3 social behavior/

4 social networks/

5 social interaction/
6 interpersonal interaction/
7 friendship/
8 exp social isolation/
9 loneliness/
10 ((social or socially) adj3 (engaged or engagement)).ti,ab,id.
11 ((social or socially) adj3 (disengaged or disengagement)).ti,ab,id.
12 ((social or socially) adj3 (connect* or disconnect*)).ti,ab,id.
13 Sociali?ation.ti,ab,id.
14 Sociali??.ti,ab,id.
15 sociali?ing.ti,ab,id.
16 Interpersonal relation?.ti,ab,id.
17 (social adj3 behavio?r*).ti,ab,id.
18 (social adj3 capital?).ti,ab,id.
19 ((social or socially) adj3 support??).ti,ab,id.
20 (social adj3 relationship?).ti,ab,id.
21 ((social or socially) adj3 participat*).ti,ab,id.
22 (social adj3 network?).ti,ab,id.
23 (social adj3 interaction*).ti,ab,id.
24 (personal adj3 network?).ti,ab,id.
25 friendship?.ti,ab,id.
26 ((social or socially) adj3 integrat*).ti,ab,id.
27 (social adj3 relation?).ti,ab,id.
28 (social adj3 contact).ti,ab,id.
29 ((social or socially) adj3 embedded*).ti,ab,id.
30 lonel*.ti,ab,id.
31 ((social or socially) adj3 isolat*).ti,ab,id.
32 (emotional* adj3 isolat*).ti,ab,id.
33 ((perceive* or percept*) adj3 isolat*).ti,ab,id.
34 ((social* or socially) adj3 alienat*).ti,ab,id.
35 aloneness.ti,ab,id.
36 solitud*.ti,ab,id.
37 Unwanted alone.ti,ab,id.
38 or/1-37
39 nursing homes/
40 residential care institutions/
41 long term care/
42 "long-term care".ti,ab,id.
43 "longterm care".ti,ab,id.
44 LTC.ti,ab,id.

45 ((home? or facility or facilities) adj2 (retirement or assisted living or residential care or extended care)).ti,ab,id.
46 home? for the aged.ti,ab,id.
47 home? for the elderly.ti,ab,id.
48 old age home?.ti,ab,id.
49 ("old age facility" or "old age facilities").ti,ab,id.
50 (nursing adj3 (facility or facilities or residence? or center? or centre?)).ti,ab,id. (1866)
51 nursing home?.ti,ab,id.
52 (residence? adj2 ('assisted living' or convalescen* or retire???? or 'long stay' or longstay or 'long term')).ti,ab,id.
53 ((facility or facilities) adj2 ('assisted living' or convalescen* or retire???? or resident* or 'long stay' or longstay or 'long term')).ti,ab,id.
54 (home? adj2 ('assisted living' or convalescen* or retire???? or 'long stay' or longstay or 'long term')).ti,ab,id.
55 "care home?".ti,ab,id.
56 (housing adj3 (retirement or old age or senior* or elderly)).ti,ab,id.
57 or/39-56
58 38 and 57
59 ("Psychometrics & Statistics & Methodology" or "Research Methods & Experimental Design").cc.
60 psychometr*.ti,ab.
61 (clinimetr* or clinometr*).ti,ab.
62 outcome assessment.ti,ab.
63 outcome measure*.ti,ab.
64 observer variation.ti,ab.
65 reproducib*.ti,ab.
66 (reliab* or unreliab* or valid* or coefficient or homogeneity or homogeneous or "internal consistency").ti,ab.
67 (cronbach* and (alpha or alphas)).ti,ab.
68 (item and (correlation* or selection* or reduction*)).ti,ab.
69 (agreement or precision or imprecision or "precise values" or test-retest).ti,ab.
70 (test and retest).ti,ab.
71 (reliab* and (test or retest)).ti,ab.
72 (stability or interrater or inter-rater or intrarater or intra-rater or intertester or inter-tester or intratester or intra-tester or interobserver or inter-observer or intraobserver or intraobserver or intertechnician or inter-technician or intratechnician or intra-technician or interexaminer or inter-examiner or intraexaminer or intra-examiner or interassay or interassay or intraassay or intra-assay or interindividual or inter-individual or intraindividual or intra-individual or interparticipant or inter-participant or intraparticipant or intra-participant or kappa or kappa's or kappas or repeatab*).ti,ab.

73 ((replicab* or repeated) and (measure or measures or findings or result or results or test or tests)).ti,ab.
74 (generaliza* or generalisa* or concordance).ti,ab.
75 (intraclass and correlation*).ti,ab.
76 (discriminative or "known group" or factor analysis or factor analyses or dimension* or subscale*).ti,ab.
77 (multitrait and scaling and (analysis or analyses)).ti,ab.
78 (item discriminant or interscale correlation* or error or errors or "individual variability").ti,ab.
79 (variability and (analysis or values)).ti,ab.
80 (uncertainty and (measurement or measuring)).ti,ab.
81 ("standard error of measurement" or sensitiv* or responsive*).ti,ab.
82 ((minimal or minimally or clinical or clinically) and (important or significant or detectable) and (change or difference)).ti,ab.
83 (small* and (real or detectable) and (change or difference)).ti,ab.
84 (meaningful change or "ceiling effect" or "floor effect" or "Item response model" or IRT or Rasch or "Differential item functioning" or DIF or "computer adaptive testing" or "item bank" or "cross-cultural equivalence").ti,ab.
85 exp measurement/
86 exp "error analysis"/
87 exp "test construction"/
88 exp "interrater reliability"/
89 exp "content analysis"/
90 exp " error of measurement"/
91 exp "factor structure"/
92 exp "testing methods"/
93 exp "statistical reliability"/
94 exp "consistency (measurement)"/
95 exp "computer assisted testing"/
96 exp "factor analysis"/
97 exp prediction/
98 exp "statistical validity"/
99 exp "prediction errors"/
100 or/59-99
101 58 and 100
102 limit 101 to ("0200 book" or "0240 authored book" or "0280 edited book" or "0300 encyclopedia" or "0400 dissertation abstract")
103 limit 101 to (abstract collection or encyclopedia entry or review-book)
104 102 or 103
105 101 not 104

106 limit 105 to (childhood <birth to 12 years> or adolescence <13 to 17 years>)
107 limit 105 to adulthood <18+ years>
108 105 not (106 not 107)

Database: CINAHL Complete
Interface: EBSCOhost
Latest search date: November 18 2021

#	Query	Limiters/Expanders
S1	(MH "Interpersonal Relations")	Search modes - Boolean/Phrase
S2	(MH "Social Participation")	Search modes - Boolean/Phrase
S3	(MH "Social Behavior")	Search modes - Boolean/Phrase
S4	(MH "Social Isolation+")	Search modes - Boolean/Phrase
S5	((social or socially) n2 (engaged or engagement))	Search modes - Boolean/Phrase
S6	((social or socially) n2 (disengaged or disengagement))	Search modes - Boolean/Phrase
S7	((social or socially) n2 (connect* or disconnect*)	Search modes - Boolean/Phrase
S8	"sociali?ation"	Search modes - Boolean/Phrase
S9	"Socialize" or "Socialise"	Search modes - Boolean/Phrase
S10	"sociali?ing"	Search modes - Boolean/Phrase
S11	Interpersonal relation OR Interpersonal relations	Search modes - Boolean/Phrase
S12	(social n2 behavio#r*)	Search modes - Boolean/Phrase

S1 3	(social w2 capital) OR (social w2 capitals)	Search modes - Boolean/Phrase
S1 4	((social or socially) w2 support*)	Search modes - Boolean/Phrase
S1 5	(social n2 relationship) OR (social n2 relationships)	Search modes - Boolean/Phrase
S1 6	((social or socially) n2 participat*)	Search modes - Boolean/Phrase
S1 7	(social n2 network) OR (social n2 networks)	Search modes - Boolean/Phrase
S1 8	(social n2 interaction*)	Search modes - Boolean/Phrase
S1 9	(personal n2 network) OR (personal n2 networks)	Search modes - Boolean/Phrase
S2 0	friendship or friendships	Search modes - Boolean/Phrase
S2 1	((social or socially) n2 integrat*)	Search modes - Boolean/Phrase
S2 2	(social n2 relation) OR (social n2 relations)	Search modes - Boolean/Phrase
S2 3	(social n2 contact)	Search modes - Boolean/Phrase
S2 4	((social or socially) n2 embedded*)	Search modes - Boolean/Phrase
S2 5	lonel*	Search modes - Boolean/Phrase
S2 6	((social or socially) n2 isolat*)	Search modes - Boolean/Phrase
S2 7	(emotional* n2 isolat*)	Search modes - Boolean/Phrase

S28	((perceive* or percept*) n2 isolat*)	Search modes - Boolean/Phrase
S29	((social* or socially) n2 alienat*)	Search modes - Boolean/Phrase
S30	aloneness	Search modes - Boolean/Phrase
S31	solitud*	Search modes - Boolean/Phrase
S32	Unwanted alone	Search modes - Boolean/Phrase
S33	S1 OR S2 OR S3 OR S4 OR S5 OR S6 OR S7 OR S8 OR S9 OR S10 OR S11 OR S12 OR S13 OR S14 OR S15 OR S16 OR S17 OR S18 OR S19 OR S20 OR S21 OR S22 OR S23 OR S24 OR S25 OR S26 OR S27 OR S28 OR S29 OR S30 OR S31 OR S32	Search modes - Boolean/Phrase
S34	(MH "Nursing Homes")	Search modes - Boolean/Phrase
S35	(MH "Nursing Home Patients")	Search modes - Boolean/Phrase
S36	(MH "Residential Facilities")	Search modes - Boolean/Phrase
S37	(MH "Long Term Care")	Search modes - Boolean/Phrase
S38	"long-term care"	Search modes - Boolean/Phrase
S39	"longterm care"	Search modes - Boolean/Phrase
S40	LTC	Search modes - Boolean/Phrase

S41	((home or homes or facility or facilities) n1 (retirement or assisted living or residential care or extended care))	Search modes - Boolean/Phrase
S42	home for the aged OR homes for the aged	Search modes - Boolean/Phrase
S43	home for the elderly OR homes for the elderly	Search modes - Boolean/Phrase
S44	old age home OR old age homes	Search modes - Boolean/Phrase
S45	("old age facility" or "old age facilities")	Search modes - Boolean/Phrase
S46	(nursing n2 (facility or facilities or residence or residences or center or centers or centre or centres))	Search modes - Boolean/Phrase
S47	"nursing home" OR "nursing homes"	Search modes - Boolean/Phrase
S48	((residence or residences) n2 ('assisted living' or convalescen* or retire or retirement or 'long stay' or longstay or 'long term'))	Search modes - Boolean/Phrase
S49	((facility or facilities) n2 ('assisted living' or convalescen* or retire or retirement or resident* or 'long stay' or longstay or 'long term'))	Search modes - Boolean/Phrase
S50	((home or homes) n2 ('assisted living' or convalescen* or retire or retirement or 'long stay' or longstay or 'long term'))	Search modes - Boolean/Phrase
S51	"care home" OR "care homes"	Search modes - Boolean/Phrase
S52	(housing n2 (retirement or old age or senior* or elderly)	Search modes - Boolean/Phrase
S53	S34 OR S35 OR S36 OR S37 OR S38 OR S39 OR S40 OR S41 OR S42 OR S43 OR S44 OR S45 OR S46 OR S47 OR S48 OR S49 OR S50 OR S51 OR S52	Search modes - Boolean/Phrase

S54	(MH “Psychometrics”) or (TI psychometr* or AB psychometr*) or (TI clinimetr* or AB clinimetr*) or (TI clinometr* OR AB clinometr*) or (MH “Outcome Assessment”) or (TI outcome assessment or AB outcome assessment) or (TI outcome measure* or AB outcome measure*) or (MH “Health Status Indicators”) or (MH “Reproducibility of Results”) or (MH “Discriminant Analysis”) or ((TI reproducib* or AB reproducib*) or (TI reliab* or AB reliab*) or (TI unreliab* or AB unreliab*)) or ((TI valid* or AB valid*) or (TI coefficient or AB coefficient) or (TI homogeneity or AB homogeneity)) or (TI homogeneous or AB homogeneous) or (TI “coefficient of variation” or AB “coefficient of variation”) or (TI “internal consistency” or AB “internal consistency”) or (MH “Internal Consistency+”) or (MH “Reliability+”) or (MH “Measurement Error+”) or (MH “Content Validity+”) or “hypothesis testing” or “structural validity” or “cross-cultural validity” or (MH “Criterion-Related Validity+”) or “responsiveness” or “interpretability” or (TI reliab* or AB reliab*) and ((TI test or AB test) OR (TI retest or AB retest)) or (TI stability or AB stability) or (TI interrater or AB interrater) or (TI inter-rater or AB inter-rater) or (TI intrarater or AB intrarater) or (TI intra-rater or AB intrarater) or (TI intertester or AB intertester) or (TI inter-tester or AB inter-tester) or (TI intratester or AB intratester) or (TI intra-tester or AB intra-tester) or (TI interobserver or AB interobserver) or (TI inter-observer or AB inter-observer) or (TI intraobserver or AB intraobserver) or (TI intra-observer or AB intra-observer) or (TI intertechnician or AB intertechnician) or (TI inter-technician or AB inter-technician) or (TI intratechnician or AB intratechnician) or (TI intra-technician or AB intra-technician) or (TI interexaminer or AB interexaminer) or (TI inter-examiner or AB inter-examiner) or (TI	Expanders - Apply equivalent subjects<br>Search modes - Boolean/Phrase

	intraexaminer or AB intraexaminer) OR (TI intra-examiner or AB intra-examiner) or (TI intra-examiner or AB intraexaminer) or (TI interassay or AB interassay) or (TI inter-assay or AB inter-assay) or (TI intraassay or AB intraassay) or (TI intra-assay or AB intra-assay) or (TI interindividual or AB interindividual) or (TI inter-individual or AB inter-individual) OR (TI intraindividual or AB intraindividual) or (TI intra-individual or AB intra-individual) or (TI interparticipant or AB interparticipant) or (TI inter-participant or AB inter-participant) or (TI intraparticipant or AB intraparticipant) or (TI intra-participant or AB intra-participant) or (TI kappa or AB kappa) or (TI kappa’s or AB kappa’s) or (TI kappas or AB kappas) or (TI repeatab* or AB repeatab*) or (TI responsive* or AB responsive*) or (TI interpretab* or AB interpretab*)	

S55	S33 AND S53 AND S54	Limiters - Publication Type: Journal Article<br>Expanders - Apply equivalent subjects<br>Search modes - Boolean/Phrase

Database: AgeLine
Interface: EBSCOhost
Latest search date: November 18 2021

#	Query	Limiters/Expanders
S1	(((DE "Interpersonal Relations") OR (DE "Isolation")) OR (DE "Socialization")) OR (DE "Loneliness")	Expanders - Apply equivalent subjects<br>Search modes - Boolean/Phrase
S2	((social or socially) n2 (engaged or engagement))	Expanders - Apply equivalent subjects<br>Search modes - Boolean/Phrase
S3	((social or socially) n2 (disengaged or disengagement))	Expanders - Apply equivalent subjects<br>Search modes - Boolean/Phrase
S4	((social or socially) n2 (connect* or disconnect*)	Expanders - Apply equivalent subjects<br>Search modes - Boolean/Phrase
S5	"sociali?ation"	Expanders - Apply equivalent subjects<br>Search modes - Boolean/Phrase
S6	"Socialize" or "Socialise"	Expanders - Apply equivalent subjects<br>Search modes - Boolean/Phrase
S7	"sociali?ing"	Expanders - Apply equivalent subjects<br>Search modes - Boolean/Phrase

S8	Interpersonal relation OR Interpersonal relations	Expanders - Apply equivalent subjects<br>Search modes - Boolean/Phrase
S9	(social n2 behavio#r*)	Expanders - Apply equivalent subjects<br>Search modes - Boolean/Phrase
S10	(social w2 capital) OR (social w2 capitals)	Expanders - Apply equivalent subjects<br>Search modes - Boolean/Phrase
S11	((social or socially) w2 support*)	Expanders - Apply equivalent subjects<br>Search modes - Boolean/Phrase
S12	(social n2 relationship) OR (social n2 relationships)	Expanders - Apply equivalent subjects<br>Search modes - Boolean/Phrase
S13	((social or socially) n2 participat*)	Expanders - Apply equivalent subjects<br>Search modes - Boolean/Phrase
S14	(social n2 network) OR (social n2 networks)	Expanders - Apply equivalent subjects<br>Search modes - Boolean/Phrase
S15	(social n2 interaction*)	Expanders - Apply equivalent subjects<br>Search modes - Boolean/Phrase
S16	(personal n2 network) OR (personal n2 networks)	Expanders - Apply equivalent subjects<br>Search modes - Boolean/Phrase
S17	friendship or friendships	Expanders - Apply equivalent subjects<br>Search modes - Boolean/Phrase
S18	((social or socially) n2 integrat*)	Expanders - Apply equivalent subjects<br>Search modes - Boolean/Phrase

S19	(social n2 relation) OR (social n2 relations)	Expanders - Apply equivalent subjects<br>Search modes - Boolean/Phrase
S20	(social n2 contact)	Expanders - Apply equivalent subjects<br>Search modes - Boolean/Phrase
S21	((social or socially) n2 embedded*)	Expanders - Apply equivalent subjects<br>Search modes - Boolean/Phrase
S22	lonel*	Expanders - Apply equivalent subjects<br>Search modes - Boolean/Phrase
S23	((social or socially) n2 isolat*)	Expanders - Apply equivalent subjects<br>Search modes - Boolean/Phrase
S24	(emotional* n2 isolat*)	Expanders - Apply equivalent subjects<br>Search modes - Boolean/Phrase
S25	((perceive* or percept*) n2 isolat*)	Expanders - Apply equivalent subjects<br>Search modes - Boolean/Phrase
S26	((social* or socially) n2 alienat*)	Expanders - Apply equivalent subjects<br>Search modes - Boolean/Phrase
S27	aloneness	Expanders - Apply equivalent subjects<br>Search modes - Boolean/Phrase
S28	solitud*	Expanders - Apply equivalent subjects<br>Search modes - Boolean/Phrase
S29	Unwanted alone	Expanders - Apply equivalent subjects<br>Search modes - Boolean/Phrase

S30	S1 OR S2 OR S3 OR S4 OR S5 OR S6 OR S7 OR S8 OR S9 OR S10 OR S11 OR S12 OR S13 OR S14 OR S15 OR S16 OR S17 OR S18 OR S19 OR S20 OR S21 OR S22 OR S23 OR S24 OR S25 OR S26 OR S27 OR S28 OR S29	Expanders - Apply equivalent subjects<br>Search modes - Boolean/Phrase
S31	((DE "Nursing Homes" OR DE "For Profit Nursing Homes" OR DE "Nonprofit Nursing Homes" OR DE "Teaching Nursing Homes") OR (DE "Homes for the Elderly")) OR (DE "Long Term Care")	Expanders - Apply equivalent subjects<br>Search modes - Boolean/Phrase
S32	"long-term care"	Expanders - Apply equivalent subjects<br>Search modes - Boolean/Phrase
S33	"longterm care"	Expanders - Apply equivalent subjects<br>Search modes - Boolean/Phrase
S34	LTC	Expanders - Apply equivalent subjects<br>Search modes - Boolean/Phrase
S35	((home or homes or facility or facilities) n1 (retirement or assisted living or residential care or extended care))	Expanders - Apply equivalent subjects<br>Search modes - Boolean/Phrase
S36	home for the aged OR homes for the aged	Expanders - Apply equivalent subjects<br>Search modes - Boolean/Phrase
S37	home for the elderly OR homes for the elderly	Expanders - Apply equivalent subjects<br>Search modes - Boolean/Phrase
S38	old age home OR old age homes	Expanders - Apply equivalent subjects<br>Search modes - Boolean/Phrase
S39	("old age facility" or "old age facilities")	Expanders - Apply equivalent subjects<br>Search modes - Boolean/Phrase

S40	(nursing n2 (facility or facilities or residence or residences or center or centers or centre or centres))	Expanders - Apply equivalent subjects<br>Search modes - Boolean/Phrase
S41	"nursing home" OR "nursing homes"	Expanders - Apply equivalent subjects<br>Search modes - Boolean/Phrase
S42	((residence or residences) n2 ('assisted living' or convalescen* or retire or retirement or 'long stay' or longstay or 'long term'))	Expanders - Apply equivalent subjects<br>Search modes - Boolean/Phrase
S43	((facility or facilities) n2 ('assisted living' or convalescen* or retire or retirement or resident* or 'long stay' or longstay or 'long term'))	Expanders - Apply equivalent subjects<br>Search modes - Boolean/Phrase
S44	((home or homes) n2 ('assisted living' or convalescen* or retire or retirement or 'long stay' or longstay or 'long term'))	Expanders - Apply equivalent subjects<br>Search modes - Boolean/Phrase
S45	"care home" OR "care homes"	Expanders - Apply equivalent subjects<br>Search modes - Boolean/Phrase
S46	(housing n2 (retirement or old age or senior* or elderly)	Expanders - Apply equivalent subjects<br>Search modes - Boolean/Phrase
S47	S31 OR S32 OR S33 OR S34 OR S35 OR S36 OR S37 OR S38 OR S39 OR S40 OR S41 OR S42 OR S43 OR S44 OR S45 OR S46	Expanders - Apply equivalent subjects<br>Search modes - Boolean/Phrase
S48	DE "Test Validity"	Expanders - Apply equivalent subjects<br>Search modes - Boolean/Phrase
S49	(TI psychometr* or AB psychometr*) or (TI clinimetr* or AB clinimetr*) or (TI clinometr* OR AB clinometr*)	Expanders - Apply equivalent subjects<br>Search modes - Boolean/Phrase
S50	(TI outcome assessment or AB outcome assessment) or (TI outcome measure* or AB outcome measure*)	Expanders - Apply equivalent subjects<br>Search modes - Boolean/Phrase

S51	(TI reproducib* or AB reproducib*) or (TI reliab* or AB reliab*) or (TI unreliab* or AB unreliab*) or (TI valid* or AB valid*) or (TI coefficient or AB coefficient) or (TI homogeneity or AB homogeneity) or (TI homogeneous or AB homogeneous) or (TI "coefficient of variation" or AB "coefficient of variation") or (TI "internal consistency" or AB "internal consistency")	Expanders - Apply equivalent subjects<br>Search modes - Boolean/Phrase
S52	"hypothesis testing" or "structural validity" or "cross-cultural validity"	Expanders - Apply equivalent subjects<br>Search modes - Boolean/Phrase
S53	"responsiveness" or "interpretability" or (TI reliab* or AB reliab*) and ((TI test or AB test) OR (TI retest or AB retest)) or (TI stability or AB stability) or (TI interrater or AB interrater) or (TI inter-rater or AB inter-rater) or (TI intrarater or AB intrarater) or (TI intra-rater or AB intrarater) or (TI intertester or AB intertester) or (TI inter-tester or AB inter-tester) or (TI intratester or AB intratester) or (TI intra-tester or AB intra-tester) or (TI interobserver or AB interobserver) or (TI inter-observer or AB inter-observer) or (TI intraobserver or AB intraobserver) or (TI intra-observer or AB intra-observer) or (TI intertechnician or AB intertechnician) or (TI inter-technician or AB inter-technician) or (TI intratechnician or AB intratechnician) or (TI intra-technician or AB intra-technician) or (TI interexaminer or AB interexaminer) or (TI inter-examiner or AB inter-examiner) or (TI intraexaminer or AB intraexaminer) OR (TI intra-examiner or AB intra-examiner) or (TI intra-examiner or AB intraexaminer) or (TI interassay or AB interassay) or (TI inter-assay or AB inter-assay) or (TI intraassay or AB intraassay) or (TI intra-assay or AB intra-assay) or (TI interindividual or	Expanders - Apply equivalent subjects<br>Search modes - Boolean/Phrase

	AB interindividual) or (TI inter-individual or AB inter-individual) OR (TI intraindividual or AB intraindividual) or (TI intra-individual or AB intra-individual) or (TI interparticipant or AB interparticipant) or (TI inter-participant or AB inter-participant) or (TI intraparticipant or AB intraparticipant) or (TI intra-participant or AB intra-participant) or (TI kappa or AB kappa) or (TI kappa’s or AB kappa’s) or (TI kappas or AB kappas) or (TI repeatab* or AB repeatab*) or (TI responsive* or AB responsive*) or (TI interpretab* or AB interpretab*)	
S54	S48 OR S49 OR S50 OR S51 OR S52 OR S53	Expanders - Apply equivalent subjects<br>Search modes - Boolean/Phrase
S55	S30 AND S47 AND S54	Limiters - Publication Type: Journal Article<br>Expanders - Apply equivalent subjects<br>Search modes - Boolean/Phrase

Database: Scopus
Interface: N/A
Latest search date: November 18 2021

(((TITLE-ABS-KEY(((social or socially) W/2 (engaged or engagement)))) OR (TITLE-ABS-KEY(((social or socially) W/2 (disengaged or disengagement)))) OR (TITLE-ABS-KEY(((social or socially) W/2 (connect* or disconnect*)))) OR (TITLE-ABS-KEY(sociali?ation)) OR (TITLE-ABS-KEY("Socialize" or "Socialise")) OR (TITLE-ABS-KEY(sociali?ing)) OR (TITLE-ABS-KEY("Interpersonal relation" OR "Interpersonal relations")) OR (TITLE-ABS-KEY((social W/2 behavio*r*))) OR (TITLE-ABS-KEY((social W/2 capital) OR (social W/2 capitals))) OR (TITLE-ABS-KEY(((social or socially) W/2 support*))) OR (TITLE-ABS-KEY((social W/2 relationship) OR (social W/2 relationships))) OR (TITLE-ABS-KEY(((social or socially) W/2 participat*))) OR (TITLE-ABS-KEY((social W/2 network) OR (social W/2 networks))) OR (TITLE-ABS-KEY((social W/2 interaction*))) OR (TITLE-ABS-KEY((personal W/2 network) OR (personal W/2 networks))) OR (TITLE-ABS-KEY(friendship or friendships)) OR (TITLE-ABS-KEY(((social or socially) W/2 integrat*))) OR (TITLE-ABS-KEY((social W/2 relation) OR (social W/2 relations))) OR (TITLE-ABS-KEY((social W/2 contact))) OR (TITLE-ABS-KEY(((social or socially) W/2 embedded*))) OR (TITLE-ABS-KEY(lonel*)) OR (TITLE-ABS-KEY(((social or socially) W/2 isolat*))) OR (TITLE-ABS-KEY((emotional* W/2 isolat*))) OR (TITLE-ABS-KEY(((perceive* or percept*) W/2 isolat*))) OR (TITLE-ABS-KEY(((social* or socially) W/2 alienat*))) OR (TITLE-ABS-KEY(aloneness)) OR (TITLE-ABS-KEY(solitud*)) OR (TITLE-ABS-KEY(Unwanted alone))) AND ((TITLE-ABS-KEY("long term care" OR "longterm care" OR LCT)) OR (TITLE-ABS-KEY((home* W/2 (retirement or "assisted living" or "residential care" or "extended care")))) OR (TITLE-ABS-KEY((facility W/2 (retirement or "assisted living" or "residential care" or "extended care")))) OR (TITLE-ABS-KEY((facilities W/2 (retirement or "assisted living" or "residential care" or "extended care")))) OR (TITLE-ABS-KEY("home* for the aged")) OR (TITLE-ABS-KEY("home* for the elderly")) OR (TITLE-ABS-KEY("old age home*")) OR (TITLE-ABS-KEY(("old age facility" or "old age facilities"))) OR (TITLE-ABS-KEY((nursing W/2 (facility or facilities or residence or residences or center or centers or centre or centres)))) OR (TITLE-ABS-KEY("nursing home" OR "nursing homes")) OR (TITLE-ABS-KEY(((residence or residences) W/2 ("assisted living" or convalescen* or retire or retirement or "long stay" or longstay or "long term")))) OR (TITLE-ABS-KEY(((facility or facilities) W/2 ("assisted living" or convalescen* or retire or retirement or "long stay" or longstay or "long term")))) OR (TITLE-ABS-KEY(((home or homes) W/2 ("assisted living" or convalescen* or retire or retirement or "long stay" or longstay or "long term")))) OR (TITLE-ABS-KEY("care home" OR "care homes")) OR (TITLE-ABS-KEY((housing W/2 (retirement or "old age" or senior* or elderly))))) AND NOT INDEX(medline)) AND (TITLE-ABS-KEY(psychometr* OR clinimetr* or clinometr* OR "outcome assessment" OR "outcome measure*" OR reproducib* OR reliab* OR unreliab* OR valid* OR coefficient OR homogeneity OR homogeneous OR "coefficient of variation" OR "internal consistency" OR "hypothesis testing" OR "structural validity" OR "cross-cultural validity" OR responsiveness OR interpretability OR reliab* OR test OR retest OR stability OR stability OR interrater OR "inter-rater" OR intrarater OR "intra-rater" OR intertester OR "inter-tester" OR intratester OR "intra-tester" OR interobserver OR "inter-observer" OR

intraobserver OR intraobserver OR intertechnician OR "inter-technician" OR intratechnician OR "intra-technician" OR interexaminer OR "inter-examiner" OR intraexaminer OR "intra-examiner" OR interassay OR interassay OR intraassay OR "intra-assay" OR interindividual OR "inter-individual" OR intraindividual OR "intra-individual" OR interparticipant OR "inter-participant" OR intraparticipant OR "intra-participant" OR kappa OR "kappa's" OR kappas OR repeatab* OR responsive* OR interpretab*)) AND (EXCLUDE (DOCTYPE,"ch") OR EXCLUDE (DOCTYPE,"bk") OR EXCLUDE (DOCTYPE,"cr"))

Database: Sociological Abstracts
Interface: Proquest
Latest search date: November 18 2021
Set#: S1
Searched for: noft(((social OR socially) n2 (disengaged OR disengagement))) OR noft(((social or socially) n2 (disengaged or disengagement))) OR noft(((social or socially) n2 (connect* or disconnect*))) OR noft(Interpersonal relation*) OR noft(Socialize OR Socialise OR sociali?ing OR sociali?ation) OR noft((social n2 (behavior* or behaviour*))) OR noft((social n2 (capital or capitals))) OR noft(((social or socially) n2 support*)) OR noft((social n2 (relationship or relationships))) OR noft(((social or socially) n2 participat*)) OR noft((social n2 (network or networks))) OR noft((social n2 interaction*)) OR noft((personal n2 network) OR (personal n2 networks)) OR noft(friendship or friendships) OR noft(((social or socially) n2 integrat*)) OR noft((social n2 relation) OR (social n2 relations)) OR noft((social n2 contact)) OR noft(((social or socially) n2 embedded*)) OR noft(lonel*) OR noft(((social or socially or emotional* or perceive* or percept*) n2 isolat*)) OR noft(((social* or socially) n2 alienat*)) OR noft(aloneness) OR noft(solitud*) OR noft("Unwanted alone")
Set#: S2
Searched for: noft("long term care" OR "longterm care" OR LTC OR "home for the aged" OR "homes for the aged" OR "home for the elderly" OR "homes for the elderly" OR "old age home" OR "old age homes" OR "old age facility" or "old age facilities" OR "nursing home" OR "nursing homes") OR noft(((home or homes or facility or facilities) n1 (retirement or "assisted living" or "residential care" or "extended care"))) OR noft((nursing n2 (facility or facilities or residence or residences or center or centers or centre or centres))) OR noft(((residence or residences) n2 ("assisted living" or convalescen* or retire or retirement or "long stay" or longstay or "long term"))) OR noft(((facility or facilities) n2 ("assisted living" or convalescen* or retire or retirement or resident* or "long stay" or longstay or "long term"))) OR noft(((home or homes) n2 ("assisted living" or convalescen* or retire or retirement or resident* or "long stay" or longstay or "long term"))) OR noft("care home" OR "care homes") OR noft((housing n2 (retirement or "old age" or senior* or elderly)))
Set#: S3
Searched for: NOFT(psychometr* OR clinimetr* or clinometr* OR "outcome assessment" OR "outcome measure*" OR reproducib* OR reliab* OR unreliab* OR valid* OR coefficient OR

homogeneity OR homogeneous OR "coefficient of variation" OR "internal consistency" OR "hypothesis testing" OR "structural validity" OR "cross-cultural validity" OR responsiveness OR interpretability OR reliab* OR test OR retest OR stability OR stability OR interrater OR "inter-rater" OR intrarater OR "intra-rater" OR intertester OR "inter-tester" OR intratester OR "intra-tester" OR interobserver OR "inter-observer" OR intraobserver OR intraobserver OR intertechnician OR "inter-technician" OR intratechnician OR "intra-technician" OR interexaminer OR "inter-examiner" OR intraexaminer OR "intra-examiner" OR interassay OR interassay OR intraassay OR "intra-assay" OR interindividual OR "inter-individual" OR intraindividual OR "intra-individual" OR interparticipant OR "inter-participant" OR intraparticipant OR "intra-participant" OR kappa OR "kappa's" OR kappas OR repeatab* OR responsive* OR interpretab*)
Set#: S4
Searched for: S1 AND S2 AND S3
Set#: S5
Searched for: (S1 AND S2 AND S3) AND stype.exact("Scholarly Journals")
