# Supplementary material for: Social connection measures for older adults living in long-term care homes: a systematic review protocol
Source: Syst Rev. 2024 Feb 15;13:67. doi: 10.1186/s13643-024-02468-6 (PMC10867987; doi:10.1186/s13643-024-02468-6)
Supplement: Supplementary file 3 — Additional file 3: Appendix III. [file 13643_2024_2468_MOESM3_ESM.pdf]

Appendix III: Data Extraction Instrument

Table 2. Draft of the data extraction form

		Response
**Population**	Country/countries in which the study conducted	
	Race and Ethnicity (copy and paste)	
	Inclusion criteria (copy and paste from paper)	
	Inclusion related to dementia or cognition	yes/no
	Exclusion criteria (copy and paste from paper)	
	Exclusion relation to dementia or cognition	yes/no
	Sample size (# LTC residents)	
	Sample size (# LTC home(s))	
	% female	
	Age (mean (SD)/ range)	
	Response rate	
	Dates of data collection	
**Context**	Name of measure	
	Identified/described as dementia specific	yes/no
	Identified/described as LTC specific	yes/no
	Response options	nominal, ordinal, ratio, interval
	Language of measure as tested in paper	English or other
	Mode of administration	self, proxy (family/friend/, staff, other)
	Observation period	
	Date the measure was published	
**Concept**	Subscales (if applicable)	
	Scale/subscale number of items	

	Names of items (cut and paste)	
