# Supplementary material for: Social connection measures for older adults living in long-term care homes: a systematic review protocol
Source: Syst Rev. 2024 Feb 15;13:67. doi: 10.1186/s13643-024-02468-6 (PMC10867987; doi:10.1186/s13643-024-02468-6)
Supplement: Supplementary file 4 — Additional file 4: Appendix IV. [file 13643_2024_2468_MOESM4_ESM.pdf]

Appendix IV

Table 3. Criteria for evidence of good measurement (COSMIN)

**Measurement property**	**Rating[1]**	**Criteria**
Structural validity	+	**CTT:**<br><br>CFA: CFI or TLI or comparable measure >0.95 OR RMSEA <0.06 OR SRMR <0.08[2]<br><br>**IRT/Rasch**:<br><br>No violation of unidimensionality[3]: CFI or TLI or comparable measure >0.95 OR RMSEA <0.06 OR SRMR <0.08<br><br>*AND*<br><br>no violation of local independence: residual correlations among the items after controlling for the dominant factor < 0.20 OR Q3's < 0.37<br><br>*AND*<br><br>no violation of monotonicity: adequate looking graphs OR item scalability >0.30<br><br>*AND*<br><br>adequate model fit:<br><br>IRT: $\chi 2$ >0.01<br><br>Rasch: infit and outfit mean squares ≥ 0.5 and ≤ 1.5 OR Z- standardized values > -2 and <2
	?	CTT: Not all information for '+' reported<br>IRT/Rasch: Model fit not reported
	-	Criteria for '+' not met

Internal consistency	+	At least low evidence[4] for sufficient structural validity[5] AND Cronbach's alpha(s) ≥ 0.70 for each unidimensional scale or subscale[6]
	?	Criteria for "At least low evidence[4] for sufficient structural validity[5]" not met
	-	At least low evidence[4] for sufficient structural validity[5] AND Cronbach's alpha(s) < 0.70 for each unidimensional scale or subscale[6]
Reliability	+	ICC or weighted Kappa ≥ 0.70
	?	ICC or weighted Kappa not reported
	-	ICC or weighted Kappa < 0.70
Measurement error	+	SDC or LoA < MIC[5]
	?	MIC not defined
	-	SDC or LoA > MIC[5]
Hypotheses testing for construct validity	+	The result is in accordance with the hypothesis[7]
	?	No hypothesis defined (by the review team)
	-	The result is not in accordance with the

		hypothesis[7]
Cross-cultural validity/measurement invariance	+	No important differences found between group factors (such as age, gender, language) in multiple group factor analysis OR no important DIF for group factors (McFadden's $R^2 < 0.02$)
	?	No multiple group factor analysis OR DIF analysis performed
	-	Important differences between group factors OR DIF was found
Criterion validity	+	Correlation with gold standard ≥ 0.70 OR AUC ≥ 0.70
	?	Not all information for '+' reported
	-	Correlation with gold standard < 0.70 OR AUC < 0.70
Responsiveness	+	The result is in accordance with the hypothesis7 OR AUC ≥ 0.70
	?	No hypothesis defined (by the review team)
	-	The result is not in accordance with the hypothesis7 OR AUC < 0.70

Adapted from Prinsen (48) under a Creative Commons Attribution 4.0 International License (http://creativecommons.org/licenses/by/4.0/). The criteria are updated by Prinsen (48) based on, e.g., Terwee et al. (44) and Prinsen (48).

Definitions: *AUC* area under the curve, *CFA* confirmatory factor analysis, *CFI* comparative fit index, *CTT* classical test theory, *DIF* differential item functioning, *ICC* intraclass correlation coefficient, *IRT* item response theory, *LoA* limits of agreement, *MIC* minimal important change, *RMSEA* root mean square error of approximation, *SEM* standard error of measurement, *SDC* smallest detectable change, *SRMR* standardized root mean residuals, *TLI* Tucker–Lewis index,

1. “+”= sufficient, “–”= insufficient, “?”= indeterminate

2. To rate the quality of the summary score, the factor structures should be equal across studies

3. Unidimensionality refers to a factor analysis per subscale, while structural validity refers to a factor analysis of a (multidimensional) patient-reported outcome measure

4. As defined by grading the evidence according to the GRADE approach

5. This evidence may come from different studies

6. The criteria “Cronbach alpha < 0.95” was deleted, as this is relevant in the development phase of a PROM and not when evaluating an existing PROM

7. The results of all studies should be taken together, and it should then be decided if 75% of the results are in accordance with the hypotheses
